# When Should the Differential Diagnosis of Croup Be Reconsidered?

**DOI:** 10.7759/cureus.99177

**Published:** 2025-12-14

**Authors:** Shun Taniguchi, Koji Kanno, Yusuke Ito

**Affiliations:** 1 Department of Pediatrics, Hyogo Prefectural Amagasaki General Medical Center, Amagasaki, JPN; 2 Department of Pediatric Emergency and Critical Care Medicine, Hyogo Prefectural Amagasaki General Medical Center, Amagasaki, JPN

**Keywords:** abscess, acute airway obstruction, croup, deep neck space abscess, laryngeal, pediatric deep neck space infections, persistent sore throat

## Abstract

Croup is a common pediatric illness characterized by a barking cough and inspiratory stridor, most often caused by viral infection. However, differential diagnoses should be considered when patients present with atypical symptoms or fail to respond to standard treatment. We report the case of a previously healthy three-year-old boy who presented with a three-day history of fever and a one-day history of barking cough. He was diagnosed with croup and treated with nebulized epinephrine and oral dexamethasone but showed minimal clinical improvement. His symptoms progressed to include a markedly severe sore throat and odynophagia. Contrast-enhanced CT revealed a 10-mm laryngeal lesion causing significant airway narrowing. Intubation was performed under sevoflurane anesthesia. Video laryngoscopy revealed a space-occupying lesion in the vestibular fold. Laryngeal fluid cultures confirmed *Streptococcus pyogenes*, establishing the diagnosis of a laryngeal abscess. The patient remained intubated for five days and received intravenous ampicillin/sulbactam for three weeks. Follow-up ultrasound one month later demonstrated complete resolution. In children with suspected croup who present with atypical features, such as severe sore throat, prolonged illness, or poor response to treatment, deep neck infections, including laryngeal abscess, should be considered. Early recognition and timely intervention are crucial for achieving favorable outcomes.

## Introduction

Croup, or viral laryngotracheitis, affects young children, particularly those aged six months to three years, and presents with a barking cough, inspiratory stridor, and hoarseness [1,2]. It is a common cause of acute upper airway obstruction in children [2]. Although nationwide epidemiologic data on outpatient croup visits are not available in Japan, reports from the United States indicate an annual average of more than 350,000 ED visits for croup, accounting for 1-2% of all pediatric encounters [1]. Most cases are self-limiting and respond effectively to corticosteroids and nebulized epinephrine [2,3].

Because of its high frequency and typically mild presentation, croup may be diagnosed without thorough evaluation. However, the differential diagnosis of acute upper airway obstruction in children is broad. Epiglottitis, once common and usually fatal, has become rare in the post-*Haemophilus influenzae *type b vaccine era; nevertheless, it requires urgent recognition and airway management [2]. Other important considerations include bacterial tracheitis, retropharyngeal or peritonsillar abscess, diphtheria, foreign body aspiration, and structural lesions such as subglottic stenosis, vascular rings, or laryngeal papillomas [2].

In this case, we aim to highlight the importance of maintaining a broad differential diagnosis when evaluating children who present with symptoms suggestive of croup but exhibit atypical clinical features.

## Case presentation

A previously healthy three-year-old boy was referred to the ED with a three-day history of fever and a one-day history of barking cough. Vital signs at presentation were as follows: blood pressure, 132/73 mmHg; heart rate, 180 bpm; respiratory rate, 42 breaths per minute; temperature, 38.2 °C; and oxygen saturation, 93% on 3 L/min of oxygen via a non-rebreather mask. Physical examination revealed inspiratory stridor, reduced air entry in both lungs on auscultation, and substernal retractions. The elevated blood pressure in the ED was attributed to sympathetic stimulation caused by tension, anxiety, and increased respiratory effort. Blood pressure measured 106/66 mmHg upon admission to the pediatric intensive care unit.

Laboratory testing on admission showed no remarkable abnormalities, except for a mild elevation in white blood cell count (10,100/μL) and C-reactive protein level (1.2 mg/dL).

**Figure 1:**
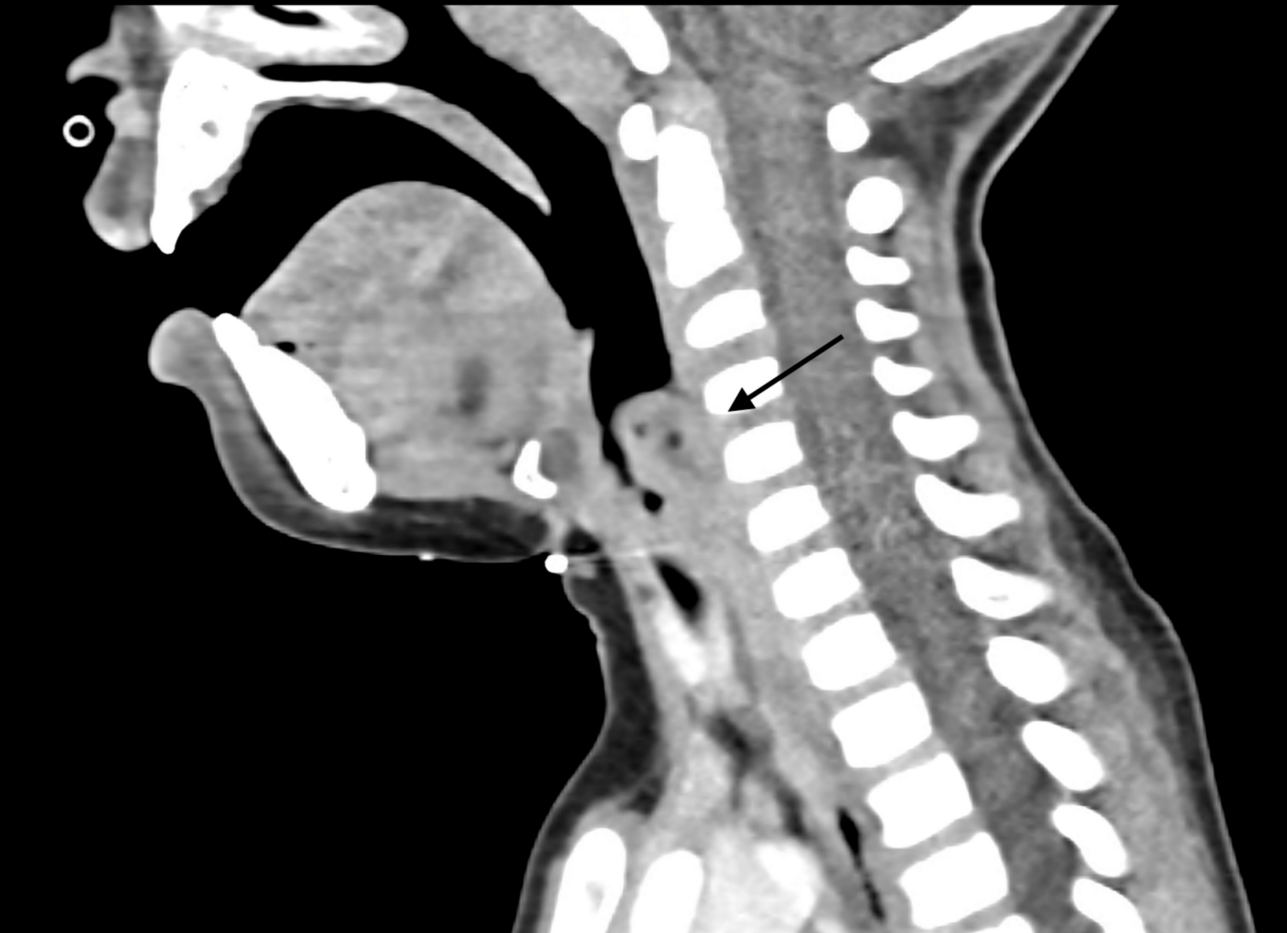
Space-occupying lesion in the larynx causing narrowing of the upper airway

The patient was initially diagnosed with croup and treated with nebulized epinephrine and oral dexamethasone. Despite receiving standard croup therapy for five days, his symptoms, particularly severe sore throat, odynophagia, and drooling, persisted, prompting consideration of an alternate diagnosis. Contrast-enhanced CT was performed due to this atypical course, revealing a space-occupying lesion in the larynx causing airway narrowing (Figure 1).

Intubation was performed to prevent exacerbation of the upper airway obstruction. Sevoflurane anesthesia was used in a controlled setting with anesthesiology and surgical backup available. Video laryngoscopy revealed a 10-mm lesion in the left false vocal cord (Video 1). *Streptococcus pyogenes* (group A) was cultured from laryngeal puncture fluid, establishing the diagnosis of laryngeal abscess.

**Video 1:** 10-mm lesion in the left false vocal cord observed via video laryngoscopy

**Figure 2:**
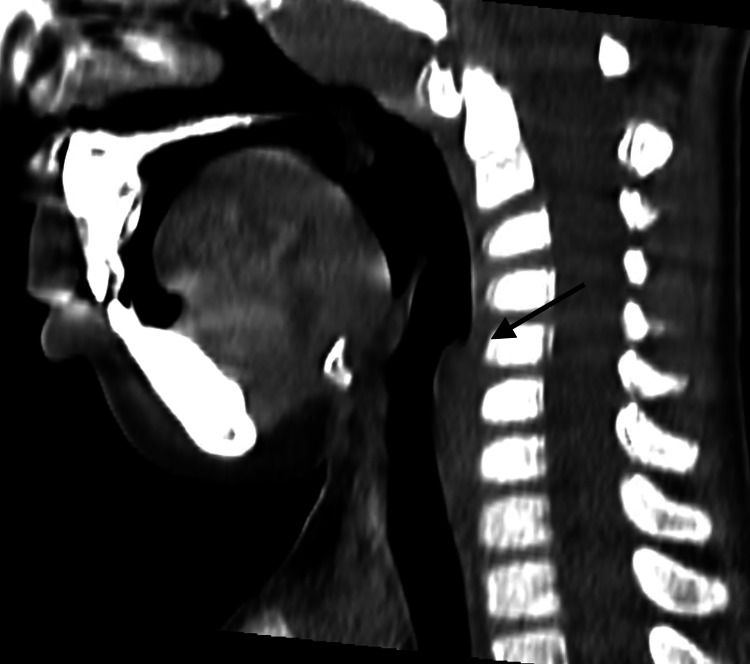
No residual or recurrent abnormalities at three months

The patient remained intubated for five days and received intravenous ampicillin/sulbactam for three weeks based on culture sensitivity. Follow-up cervical ultrasonography at one month showed complete resolution without complications. Two months after the resolution, he developed a recurrent fever and a persistent wet cough. To exclude residual or recurrent disease in a location with potential airway compromise, contrast-enhanced CT of the neck was obtained at three months, which demonstrated no abnormalities (Figure 2). 

Further microbiological analysis was performed on the isolate. PCR analysis revealed positivity for four of the five major pyrogenic exotoxin genes (speA, speB, speC, and speF) and negativity for ssa (streptococcal superantigen A). The emm type was determined to be 1.0 (Table 1). Additional SNP genotyping showed the following: rofA gene, M1global (-), M1UK (+); gldA gene, M1global (+), M1UK (-); and pstB gene, M1global (+), M1UK (-).

**Table 1:** Genetic profile of the Streptococcus pyogenes strain

Specimen	speA	speB	speC	speF	ssa	emm type
Abscess fluid	+	+	+	+	-	1.0

## Discussion

While croup typically presents with a barking cough and inspiratory stridor, several features in this case, including severe sore throat, drooling, persistence beyond the typical 24- to 72-hour course, and poor response to standard therapy, warranted further investigation. In a prospective study comparing croup and epiglottitis in children, nearly all patients with croup presented with cough and inspiratory stridor, whereas sore throat and drooling were observed in fewer than 10% of cases, suggestive of epiglottitis [4]. The median duration of cough was approximately two to three days, indicating that persistence beyond the typical 24- to 72-hour course is unusual [5-8]. This case highlights the importance of reconsidering the initial diagnosis when patients exhibit an atypical course or fail to respond to standard treatment.

Deep neck infections, including retropharyngeal and peritonsillar abscesses, are well-recognized causes of upper airway obstruction in children and should be included in the differential diagnosis of croup [2]. However, to our knowledge, laryngeal abscess in children has not been previously reported. In adults, laryngeal abscesses are exceedingly rare, accounting for only 0.4% of all deep neck abscesses, with most cases co-occurring with epiglottitis; truly spontaneous cases are considered extremely uncommon [9].

Group A *Streptococcus *(GAS) is classified based on the emm gene sequence, which encodes the M protein, a well-known virulence factor. Among these, emm1 (M1) strains are the most frequently isolated. Since 2011, the United Kingdom has reported an increasing prevalence of a distinct M1 sublineage, termed the M1UK lineage, characterized by 27 unique single-nucleotide substitutions. This lineage has subsequently become the dominant M1 sublineage in several regions, including Europe, North America, and Australia. M1UK strains produce approximately ninefold higher levels of streptococcal pyrogenic exotoxins compared with non-M1UK emm1 strains, contributing to enhanced transmissibility.

In Japan, a rise in GAS-related STSS and pharyngitis was noted in 2023, and from the summer onward, clusters of M1UK lineage strains, previously widespread in the UK during the 2010s and considered to possess increased virulence and transmissibility, were identified domestically for the first time. Because the present case involved an abscess arising in an anatomically uncommon site, additional microbiological testing was performed to determine whether the isolate possessed any distinctive genetic features. According to the National Institute of Infectious Diseases (Japan), typical *S. pyogenes* M1UK lineage strains are positive for all three markers (rofA, gldA, and pstB). Therefore, this strain cannot be categorized as a conventional UK lineage. The findings instead suggest an M1UK sublineage, given the presence of rofA SNPs, distinguishing it from the conventional M1global lineage [10]. Whether this strain is specifically associated with the development of laryngeal abscess requires further investigation.

## Conclusions

This case underscores the importance of maintaining a broad differential diagnosis when evaluating children who present with symptoms suggestive of croup but demonstrate atypical features or fail to respond to standard therapy. Deep neck infections, although uncommon, should be considered in such cases, and early imaging and airway evaluation are crucial. Laryngeal abscess remains a rare diagnosis, particularly in children, and this report highlights the need for vigilance and timely intervention to prevent critical complications.
